# Crystal structure and Hirshfeld surface analysis of the chalcone derivative (2*E*)-3-[4-(di­phenyl­amino)phen­yl]-1-[4-(prop-1-yn-2-yl­oxy)phen­yl]prop-2-en-1-one

**DOI:** 10.1107/S2056989024011721

**Published:** 2025-01-01

**Authors:** Sundarasamy Madhan, M. NizamMohideen, Vijayan Viswanathan, Devadasan Velmurugan

**Affiliations:** a Department of Physics The New College Chennai 600 014 University of Madras,Tamil Nadu India; b Centre of Excellence in Structural Biology and Drug Discovery Department of Biotechnology SRM Institute of Science and Technology, Kattankulathur Campus Chengalpattu Tamil Nadu - 603203 India; c Department of Biotechnology SRM Institute of Science and Technology, Kattankulathur Campus Chengalpattu Tamil Nadu - 603203 India; Vienna University of Technology, Austria

**Keywords:** crystal structure, chalcone, tri­phenyl­amine, hydrogen bonding, Hirshfeld surface analysis, disorder

## Abstract

In the crystal structure of the title chalcone derivative, C_30_H_23_NO_2_, the mol­ecule adopts an s-*cis* conformation with respect to the C=O and C=C bonds. The tri­phenyl­amine moiety has a propeller-type shape, with dihedral angles between the mean planes of pairs of phenyl rings of 72.1 (6), 69.7 (1) and 65.6 (6)°. In the crystal, mol­ecules are linked by C—H⋯O hydrogen bonds, forming chains extending parallel to [010]. In addition, weak C—H⋯π inter­actions consolidate the crystal packing. One of the phenyl rings of the tri­phenyl­amine moiety is disordered over two sets of sites.

## Abstract 2

The mol­ecule adopts an s-*cis* conformation with respect to the C=O and C—C bonds of the chalcone bridge.

## 1. Chemical context

Chalcones are an important class of compounds, with the common structural entity being the central –CH=CH—C(=O)– bridge, in which the H atoms can be substituted. Chalcones provide a necessary configuration for NLO activity, with two planar rings connected through a conjugated double bond (NizamMohideen *et al.*, 2007). They are also inherently chiral owing to the fact that the two phenyl rings are mutually twisted with respect to the linking backbone (Butcher *et al.*, 2006 ▸). This helicity has also been shown to lead to NLO activity (Botek *et al.*, 2004 ▸). The design of the chalcone system, *e.g*. in terms of donor—π⋯acceptor (*D*—π⋯*A*) inter­actions, plays a significant role in intra­molecular charge-transfer transitions (ICT) where optical excitation leads to the movement of charge from the donor group to the acceptor group. In addition, the chalcone bridge consists of two different double bonds, C=C and C=O, which contribute to the conjugation of charge transfer, leading to inter­esting spectroscopic properties (de Toledo *et al.*, 2018 ▸). The biological properties of chalcone derivatives such as anti­cancer (Bhat *et al.*, 2005 ▸), anti­malarial (Xue *et al.*, 2004 ▸), anti-oxidant and anti­microbial (Yayli *et al.*, 2006 ▸), anti­platelet (Zhao *et al.*, 2005 ▸) as well as anti-inflammatory (Madan *et al.*, 2000 ▸) activities have been studied extensively and are constantly developed further. Tri­phenyl­amine (TPA) derivatives, on the other hand, are important compounds used in numerous applications, *e.g.* in dye-sensitized solar cells (Lin *et al.*, 2010 ▸).
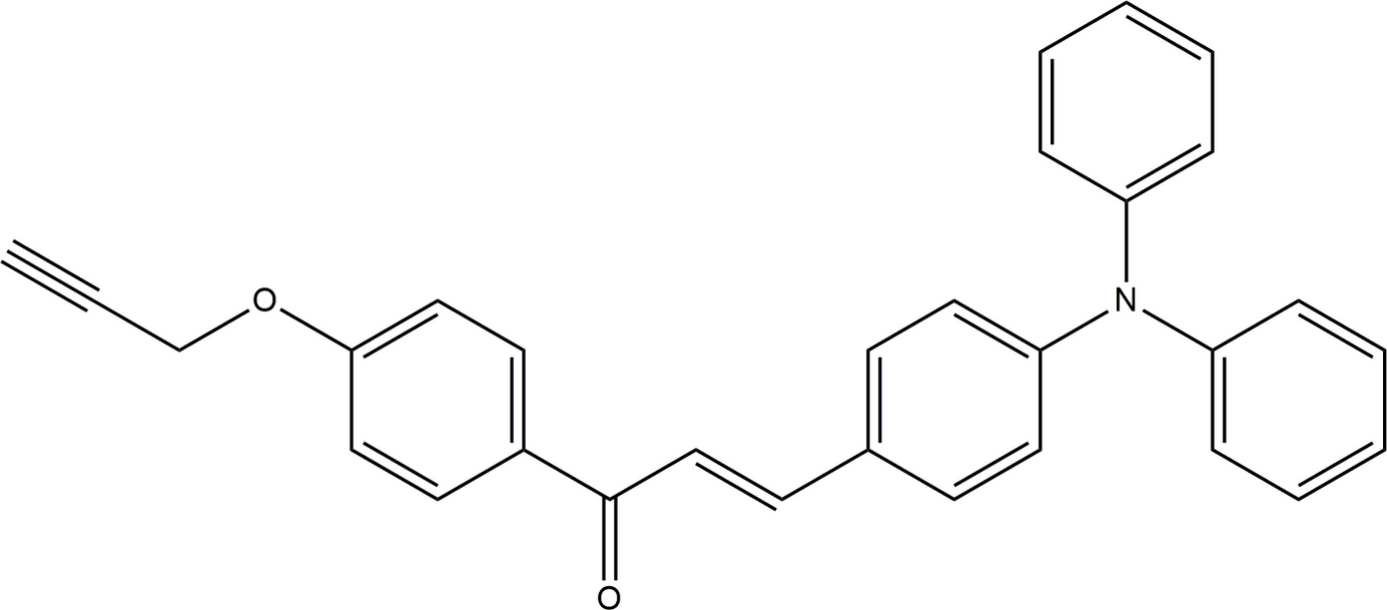


In view of the application potentials mentioned above, we synthesized the title compound and report here its mol­ecular and crystal structure and Hirshfeld surface analysis.

## 2. Structural commentary

**Figure 1:**
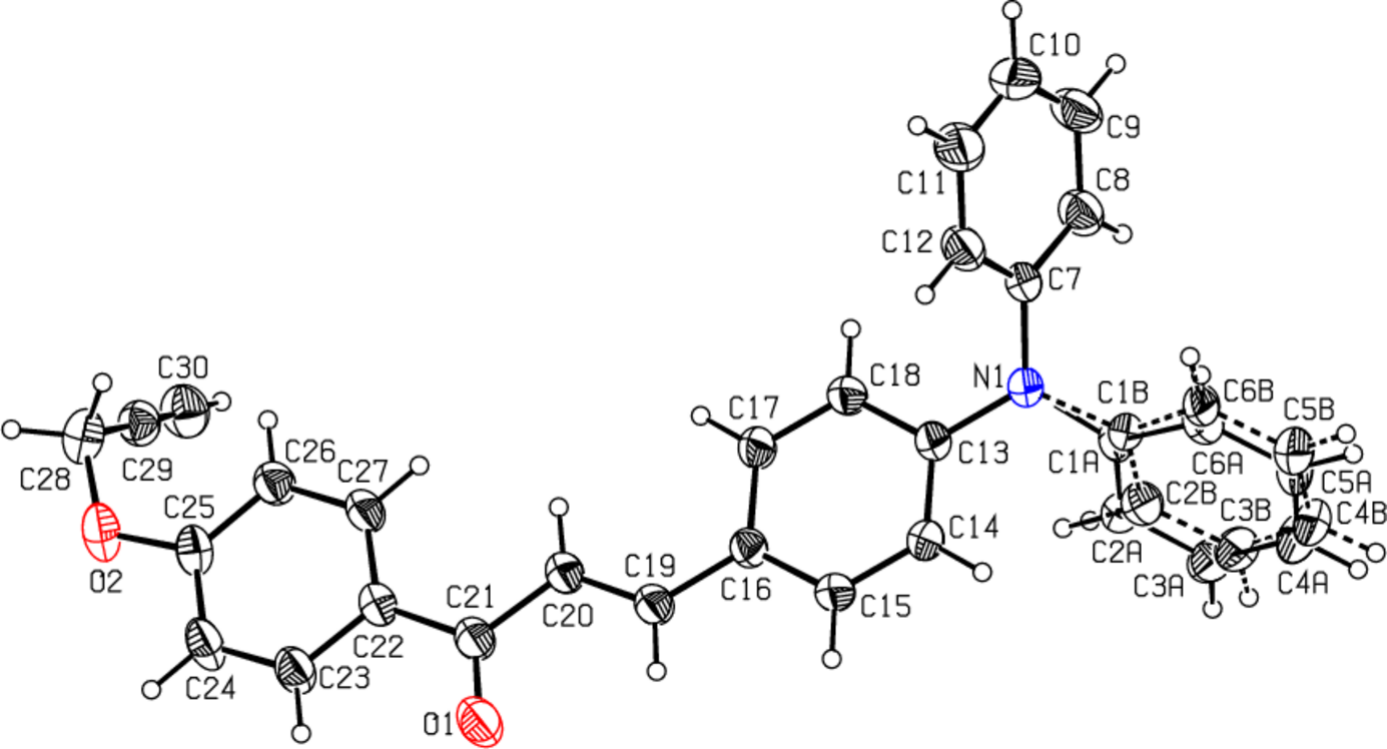
The mol­ecular structure of the title compound. Displacement ellipsoids are drawn at the 30% probability level.

In the mol­ecular structure of the title compound, Fig. 1 ▸, the enone moiety (O1/C19–C21) has a maximum deviation from planarity of 0.0217 (18) Å at C21 and adopts ans-*cis* conformation with respect to the C21=O1 bond [1.2161 (17) Å] and the C19=C20 bond [1.3230 (18) Å]. The mol­ecule is twisted about the C20—C21 bond with a C19—C20—C21—O1 torsion angle of 5.4 (2)°. A slight twist is also observed about the C21—C22 bond with an O1—C21—C22—C27 torsion angle of 179.55 (15)°. The twisted nature of this part of the mol­ecule is expected because of the steric effects between the carbonyl group and the vicinal phenyl group (Kozlowski *et al.*, 2007 ▸).

The (C1–C6) phenyl ring is disordered over two sets of siteswith a refined occupancy ratio of 0.55 (3):0.45 (3). In the tri­phenyl­amine group, the three phenyl rings form a propeller-type shape with dihedral angles of 72.1 (6)° between rings (C1–C6) and (C7–C12), of 65.6 (6)° between rings (C1—C6) and (C13–C18), and of 69.7 (1)° between rings (C7—C12) and (C13–C18). The enone moiety forms dihedral angles of 5.64 (10), 68.0 (5), 68.93 (10) and 4.18 (10)°, respectively, with the (C22–C27) phenyl ring and the (C1–C6), (C7–C12), and (C13–C18) phenyl rings of the tri­phenyl­amine group. The large variation of the dihedral angles between the enone moiety and the phenyl rings indicates that the possibility for electronic effects has decreased (Jung *et al.*, 2008 ▸). The widening of the C20—C19—C16 angle to 127.70 (13)° and of the C19—C16—C17 angle to 122.83 (12)° can be ascribed to the short inter­atomic contact between atoms H20⋯H17 (2.22 Å). In addition, the strain induced by the short H27⋯H20 (2.10 Å) contact results in a slight opening of the C21—C22—C27 angle to 123.91 (12)°. Similar features have been observed in other comparable structures (Nizam Mohideen *et al.*, 2007 ▸; Ravishankar *et al.*, 2005 ▸).

## 3. Supra­molecular features

**Figure 2:**
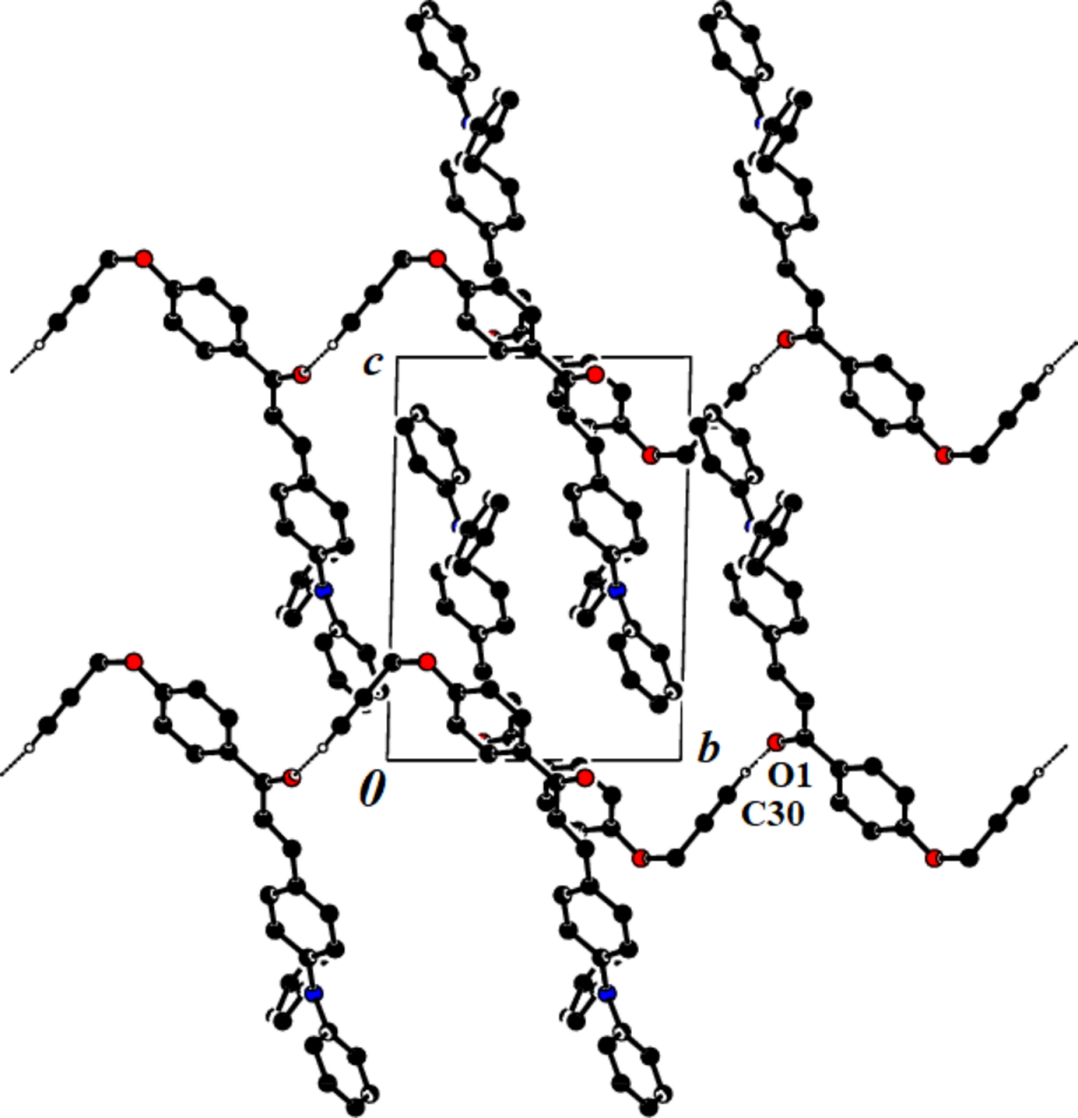
A view along the *a* axis of the title compound, showing the crystal packing. C—H⋯O hydrogen bonds are shown as dashed lines; H atoms not involved in hydrogen bonding have been omitted.

In the crystal, the mol­ecules are linked *via* weak C30—H30⋯O1 inter­molecular inter­actions (Table 1 ▸) into chains extending parallel to [010] with a *C*(11) motif (Bernstein *et al.* 1995 ▸). The crystal packing also features C—H⋯π inter­actions [C28—H28*A*⋯*Cg*1^i^ and C30—H30⋯*Cg*2^ii^, where *Cg*1 and *Cg*2 are the centres of gravity of the rings (C7–C12) and (C22—C27)]. Numerical details of the latter are compiled in Table 1 ▸, and a packing view is shown in Fig. 2 ▸.

## 4. Hirshfeld surface analysis

**Figure 3:**
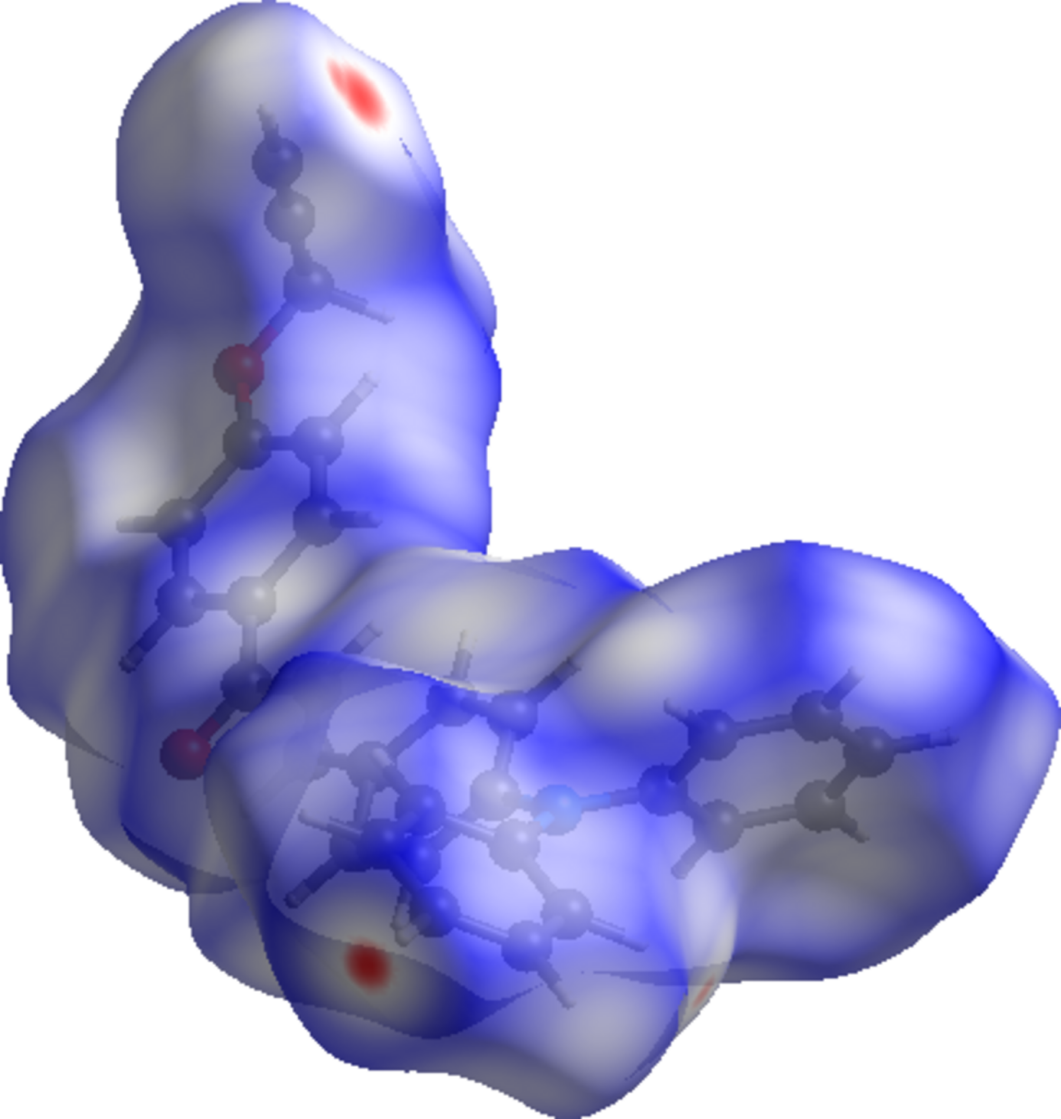
The Hirshfeld surfaces of the title compound mapped over *d*_norm_.

**Figure 4:**
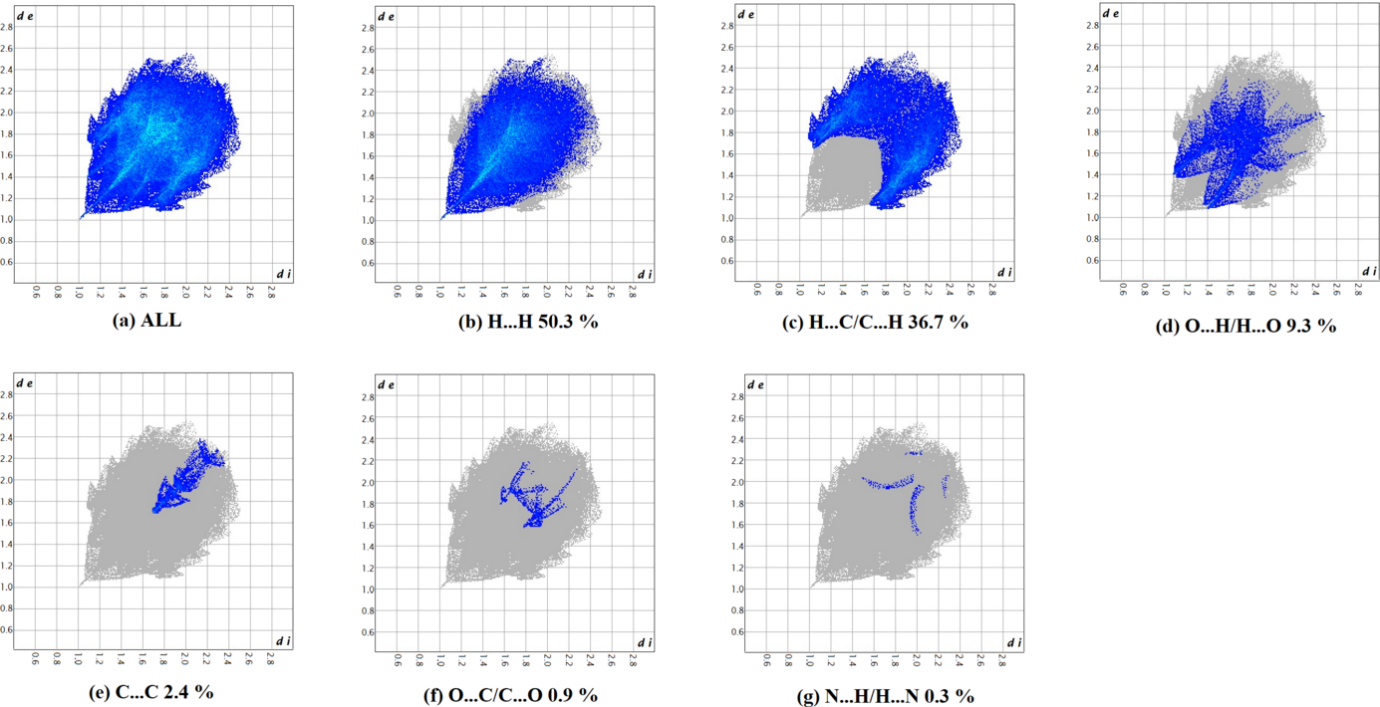
(*a*) The full two-dimensional fingerprint plot for the title compound, and fingerprint plots delineated into (*b*) H⋯H, (*c*) C⋯H/H⋯C, (*d*) O⋯H/H⋯O, (*e*) C⋯C (*f*) O⋯C/C⋯O and (*g*) N⋯H/H⋯N contacts.

A recent article by Tiekink and collaborators (Tan *et al.*, 2019 ▸) reviewed and described the utility of Hirshfeld surface analysis (Spackman & Jayatilaka, 2009 ▸) and the associated two-dimensional fingerprint plots (McKinnon *et al.*, 2007 ▸) for analysis and qu­anti­fication of inter­molecular contacts in crystals. We also performed such calculations (surface mapped over *d*_norm_ and two-dimensional fingerprint plots) by using *CrystalExplorer* (Spackman *et al.*, 2021 ▸). The Hirshfeld surface of the title compound mapped over *d*_norm_ is shown in Fig. 3 ▸, where the normalized contact distance, *d*_norm_, is colour-mapped from red (distances shorter than the sum of the van der Waals radii) through white to blue (distances longer than the sum of the van der Waals radii). The red spots visible indicate the inter­molecular contacts involved in hydrogen-bonding inter­actions, as discussed above. The two-dimensional fingerprint plots detailing the various inter­actions are displayed in Fig. 4 ▸*a* for all contacts. H⋯H inter­molecular contacts predominate, followed by the C⋯H/H⋯C and O⋯H/H⋯O contacts corresponding to the different kinds of C—H⋯π and C—H⋯O bonds. This is manifested by the contributions of H⋯H contacts at 50.3% (Fig. 4 ▸*b*), H⋯C/C⋯H contacts at 36.7% (Fig. 4 ▸*c*), and O⋯H/H⋯O at 9.3% (Fig. 4 ▸*d*). Other contacts, *viz*. C⋯C at 2.4% (Fig. 4 ▸*e*), O⋯C/C⋯O at 0.9% (Fig. 4 ▸*f*) and N⋯H/H⋯N contacts at 0.3% (Fig. 4 ▸*g*) play a minor role.

## 5. Database survey

A survey of Cambridge Structural Database (CSD, Version 5.38; Groom *et al.*, 2016 ▸) revealed fused-ring-substituted chalcones similar to the title compound. There are four compounds that have an anthracene ketone substituent on the chalcone: 9-anthryl styryl ketone and 9,10-anthryl bis­(styryl ketone) (CCDC codes: 1827021 and 1827019; Harlow *et al.*, 1975 ▸), (2*E*)-1-(anthracen-9-yl)-3-[4-(propan-2-yl)phen­yl]prop-2-en-1-one (CCDC 1494027 and 1494026; Girisha *et al.*, 2016 ▸) and (*E*)-1-(anthracen-9-yl)-3-(2-chloro-6-fluoro­phen­yl)prop-2-en-1-one (CCDC 1470351; Abdullah *et al.*, 2016 ▸). Zainuri *et al.* (2018*a* ▸,*b* ▸) reported two anthracene substituents on the chalcone (*E*)-1,3-bis­(anthracen-9-yl)prop-2-en-1-one (CCDC 1817217). Other related compounds include 1-(anthracen-9-yl)-2-methyl­prop- 2-en-1-one (CCDC 1817219; Agrahari *et al.*, 2015 ▸) and 9-anthroylacetone (CCDC 1817253; Cicogna *et al.*, 2004 ▸). For tri­phenyl­amine derivatives, Lin *et al.* (2010 ▸) reported a compound with the tri­phenyl­amine moiety in a similar propeller-type shape (CCDC 1051418), with dihedral angles between the mean planes of pairs of rings of 71.6 (2), 69.7 (1) and 65.8 (2)°, which is comparable with the title compound.

## 6. Synthesis and crystallization

To a 100 ml methanol solution of 4-(di­phenyl­amino) benzaldehyde (1.37 g, 5.0 mmol) was added 2-acetyl­pridine (0.61 g, 5.0 mmol). The mixture was stirred for 2 h at room temperature. The yellow precipitate formed was collected by filtration, and washed sequentially with water and methanol for three times, respectively. Removal of the solvent *in vacuo* followed by recrystallization from methanol (4 ml) afforded crystals of the title compound suitable for single crystal X-ray studies.

## 7. Refinement

Crystal data, data collection and structure refinement details are summarized in Table 2 ▸. All H atoms were positioned geometrically and constrained to ride on their parent atoms. The (C1–C6) phenyl ring is disordered over two sets of sites with a refined occupancy ratio of 0.55 (3):0.45 (3). The ring geometries were regularized using soft restraints.

## Supplementary Material

Crystal structure: contains datablock(s) global, I. DOI: 10.1107/S2056989024011721/wm5735sup1.cif

Structure factors: contains datablock(s) I. DOI: 10.1107/S2056989024011721/wm5735Isup2.hkl

CCDC reference: 2406922

Additional supporting information:  crystallographic information; 3D view; checkCIF report

## Figures and Tables

**Table 1:** Hydrogen-bond geometry (Å, °)

*D*—H⋯*A*	*D*—H	H⋯*A*	*D*⋯*A*	*D*—H⋯*A*
C30—H30⋯O1^i^	0.93	2.55	3.054 (2)	114
C28—H28*A*⋯*Cg*(C7–C12)^ii^	0.93	2.97	3.853 (2)	153
C30—H30⋯*Cg*(C22–C27)^iii^	0.93	2.72	3.527 (2)	145

Symmetry codes: (i) 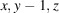; (ii) 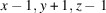; (iii) 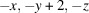.

**Table 2:** Experimental details

Crystal data	
Chemical formula	C_30_H_23_NO_2_
*M*_r_	429.49
Crystal system, space group	Triclinic, *P* 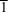
Temperature (K)	293
*a*, *b*, *c* (Å)	9.6337 (1), 9.8825 (2), 12.8446 (3)
α, β, γ (°)	87.546 (2), 86.605 (1), 70.914 (3)
*V* (Å^3^)	1153.26 (4)
*Z*	2
Radiation type	Mo *K*α
μ (mm^−1^)	0.08
Crystal size (mm)	0.29 × 0.24 × 0.20

Data collection	
Diffractometer	Bruker D8 VENTURE diffractometer with PHOTON II detector
Absorption correction	Multi-scan (*SADABS*; Krause *et al.*, 2015 ▸)
*T*_min_, *T*_max_	0.786, 0.841
No. of measured, independent and observed [*I* > 2σ(*I*)] reflections	17043, 4720, 3564
*R*_int_	0.027
(sin θ/λ)_max_ (Å^−1^)	0.627

Refinement	
*R*[*F*^2^ > 2σ(*F*^2^)], *wR*(*F*^2^), *S*	0.042, 0.120, 1.03
No. of reflections	4720
No. of parameters	353
No. of restraints	186
H-atom treatment	H-atom parameters constrained
Δρ_max_, Δρ_min_ (e Å^−3^)	0.14, −0.18

Computer programs: *APEX3* and *SAINT* (Bruker, 2016 ▸), *SHELXT* (Sheldrick, 2015*a* ▸), *SHELXL* (Sheldrick, 2015*b* ▸), *ORTEP-3 for Windows* and *WinGX* (Farrugia, 2012 ▸), *Mercury* (Macrae *et al.*, 2008 ▸), *publCIF* (Westrip, 2010 ▸) and *PLATON* (Spek, 2020 ▸).

## supplementary crystallographic information

### (2E)-3-[4-(Diphenylamino)phenyl]-1-[4-(prop-1-yn-2-yloxy)phenyl]prop-2-en-1-one Crystal data

**Table:**

C_30_H_23_NO_2_	*Z* = 2
*M**_r_* = 429.49	*F*(000) = 452
Triclinic, *P*1	*D*_x_ = 1.237 Mg m^−^^3^
*a* = 9.6337 (1) Å	Mo *K*α radiation, λ = 0.71073 Å
*b* = 9.8825 (2) Å	Cell parameters from 4720 reflections
*c* = 12.8446 (3) Å	θ = 1.6–26.5°
α = 87.546 (2)°	µ = 0.08 mm^−^^1^
β = 86.605 (1)°	*T* = 293 K
γ = 70.914 (3)°	Block, colourless
*V* = 1153.26 (4) Å^3^	0.29 × 0.24 × 0.20 mm

### (2E)-3-[4-(Diphenylamino)phenyl]-1-[4-(prop-1-yn-2-yloxy)phenyl]prop-2-en-1-one Data collection

**Table:**

Bruker D8 VENTURE diffractometer with PHOTON II detector	4720 independent reflections
Radiation source: fine-focus sealed tube	3564 reflections with *I* > 2σ(*I*)
Graphite monochromator	*R*_int_ = 0.027
ω and φ scan	θ_max_ = 26.5°, θ_min_ = 1.6°
Absorption correction: multi-scan (*SADABS*; Krause *et al.*, 2015)	*h* = −12→11
*T*_min_ = 0.786, *T*_max_ = 0.841	*k* = −12→12
17043 measured reflections	*l* = −15→16

### (2E)-3-[4-(Diphenylamino)phenyl]-1-[4-(prop-1-yn-2-yloxy)phenyl]prop-2-en-1-one Refinement

**Table:**

Refinement on *F*^2^	186 restraints
Least-squares matrix: full	Hydrogen site location: inferred from neighbouring sites
*R*[*F*^2^ > 2σ(*F*^2^)] = 0.042	H-atom parameters constrained
*wR*(*F*^2^) = 0.120	*w* = 1/[σ^2^(*F*_o_^2^) + (0.0573*P*)^2^ + 0.1568*P*] where *P* = (*F*_o_^2^ + 2*F*_c_^2^)/3
*S* = 1.03	(Δ/σ)_max_ = 0.001
4720 reflections	Δρ_max_ = 0.14 e Å^−^^3^
353 parameters	Δρ_min_ = −0.18 e Å^−^^3^

### (2E)-3-[4-(Diphenylamino)phenyl]-1-[4-(prop-1-yn-2-yloxy)phenyl]prop-2-en-1-one Special details

**Table:**

Geometry. All esds (except the esd in the dihedral angle between two l.s. planes) are estimated using the full covariance matrix. The cell esds are taken into account individually in the estimation of esds in distances, angles and torsion angles; correlations between esds in cell parameters are only used when they are defined by crystal symmetry. An approximate (isotropic) treatment of cell esds is used for estimating esds involving l.s. planes.

### (2E)-3-[4-(Diphenylamino)phenyl]-1-[4-(prop-1-yn-2-yloxy)phenyl]prop-2-en-1-one Fractional atomic coordinates and isotropic or equivalent isotropic displacement parameters (Å^2^)

**Table:**

	*x*	*y*	*z*	*U*_iso_*/*U*_eq_	Occ. (<1)
C1A	0.8450 (13)	0.3165 (18)	0.3291 (11)	0.0477 (19)	0.55 (3)
C2A	0.9929 (12)	0.2650 (13)	0.2998 (8)	0.0526 (15)	0.55 (3)
H2A	1.057776	0.201128	0.344055	0.063*	0.55 (3)
C3A	1.0457 (13)	0.3066 (13)	0.2066 (9)	0.0686 (18)	0.55 (3)
H3A	1.145916	0.271704	0.189096	0.082*	0.55 (3)
C4A	0.9533 (17)	0.3982 (14)	0.1395 (7)	0.080 (2)	0.55 (3)
H4A	0.990125	0.423346	0.075644	0.095*	0.55 (3)
C5A	0.8058 (17)	0.4535 (13)	0.1660 (7)	0.077 (2)	0.55 (3)
H5A	0.742187	0.517295	0.121020	0.092*	0.55 (3)
C6A	0.7529 (14)	0.4126 (16)	0.2612 (10)	0.063 (2)	0.55 (3)
H6A	0.653223	0.450652	0.279674	0.076*	0.55 (3)
C1B	0.8233 (15)	0.328 (2)	0.3218 (12)	0.045 (2)	0.45 (3)
C2B	0.9676 (15)	0.2842 (17)	0.2828 (11)	0.058 (2)	0.45 (3)
H2B	1.039659	0.215152	0.318986	0.070*	0.45 (3)
C3B	1.0043 (14)	0.3432 (17)	0.1894 (10)	0.066 (2)	0.45 (3)
H3B	1.100923	0.314279	0.162368	0.079*	0.45 (3)
C4B	0.8958 (17)	0.4449 (16)	0.1379 (7)	0.068 (2)	0.45 (3)
H4B	0.918844	0.485488	0.075329	0.082*	0.45 (3)
C5B	0.7523 (15)	0.4875 (14)	0.1782 (9)	0.069 (2)	0.45 (3)
H5B	0.679939	0.556822	0.142379	0.082*	0.45 (3)
C6B	0.7147 (14)	0.4297 (18)	0.2698 (11)	0.0530 (18)	0.45 (3)
H6B	0.617789	0.458367	0.296255	0.064*	0.45 (3)
C7	0.65146 (14)	0.24068 (14)	0.42815 (10)	0.0489 (3)	
C8	0.61478 (18)	0.16550 (16)	0.35241 (12)	0.0652 (4)	
H8	0.678822	0.132399	0.295076	0.078*	
C9	0.4812 (2)	0.13957 (19)	0.36246 (15)	0.0819 (5)	
H9	0.455737	0.089729	0.311064	0.098*	
C10	0.3869 (2)	0.1863 (2)	0.44679 (16)	0.0889 (6)	
H10	0.298374	0.167343	0.453336	0.107*	
C11	0.42347 (19)	0.2606 (2)	0.52096 (14)	0.0865 (6)	
H11	0.359410	0.292737	0.578450	0.104*	
C12	0.55364 (16)	0.28890 (19)	0.51205 (11)	0.0660 (4)	
H12	0.576464	0.341157	0.563074	0.079*	
C13	0.87208 (14)	0.24616 (14)	0.51006 (10)	0.0481 (3)	
C14	0.94811 (16)	0.33765 (16)	0.53177 (11)	0.0594 (4)	
H14	0.943729	0.415330	0.487056	0.071*	
C15	1.03041 (15)	0.31498 (15)	0.61902 (11)	0.0558 (3)	
H15	1.080387	0.378125	0.632154	0.067*	
C16	1.04042 (13)	0.20048 (13)	0.68753 (9)	0.0459 (3)	
C17	0.96304 (15)	0.10926 (14)	0.66508 (10)	0.0529 (3)	
H17	0.967085	0.031665	0.709799	0.063*	
C18	0.88078 (15)	0.13138 (14)	0.57822 (10)	0.0531 (3)	
H18	0.830398	0.068598	0.565046	0.064*	
C19	1.12578 (14)	0.18198 (14)	0.77995 (10)	0.0510 (3)	
H19	1.174152	0.248200	0.787474	0.061*	
C20	1.14274 (14)	0.08214 (14)	0.85454 (10)	0.0508 (3)	
H20	1.098158	0.012326	0.849705	0.061*	
C21	1.23054 (16)	0.07921 (15)	0.94488 (11)	0.0567 (3)	
C22	1.25937 (14)	−0.04205 (14)	1.02197 (10)	0.0504 (3)	
C23	1.34822 (18)	−0.04494 (16)	1.10440 (11)	0.0626 (4)	
H23	1.384847	0.030279	1.111044	0.075*	
C24	1.38253 (19)	−0.15626 (16)	1.17565 (11)	0.0671 (4)	
H24	1.442908	−0.156542	1.229593	0.081*	
C25	1.32810 (16)	−0.26811 (14)	1.16796 (10)	0.0548 (3)	
C26	1.23931 (15)	−0.26825 (15)	1.08761 (11)	0.0564 (3)	
H26	1.202191	−0.343312	1.081871	0.068*	
C27	1.20619 (15)	−0.15555 (15)	1.01576 (11)	0.0552 (3)	
H27	1.146378	−0.155960	0.961620	0.066*	
C28	1.31454 (19)	−0.49115 (16)	1.24225 (12)	0.0678 (4)	
H28A	1.208519	−0.454902	1.237709	0.081*	
H28B	1.336179	−0.545667	1.307320	0.081*	
C29	1.37787 (16)	−0.58578 (15)	1.15584 (11)	0.0583 (4)	
C30	1.4256 (2)	−0.66103 (18)	1.08611 (13)	0.0742 (4)	
H30	1.463778	−0.721228	1.030325	0.089*	
N1	0.78739 (12)	0.26872 (13)	0.42113 (8)	0.0565 (3)	
O1	1.28056 (16)	0.17550 (14)	0.95620 (10)	0.0973 (5)	
O2	1.36880 (13)	−0.37308 (11)	1.24355 (7)	0.0704 (3)	

### (2E)-3-[4-(Diphenylamino)phenyl]-1-[4-(prop-1-yn-2-yloxy)phenyl]prop-2-en-1-one Atomic displacement parameters (Å^2^)

**Table:**

	*U*^11^	*U*^22^	*U*^33^	*U*^12^	*U*^13^	*U*^23^
C1A	0.056 (4)	0.050 (3)	0.040 (3)	−0.019 (3)	−0.018 (2)	0.0096 (19)
C2A	0.055 (3)	0.054 (3)	0.052 (3)	−0.022 (2)	−0.005 (2)	0.0072 (19)
C3A	0.075 (4)	0.076 (4)	0.063 (3)	−0.037 (3)	0.006 (3)	0.001 (3)
C4A	0.101 (6)	0.092 (5)	0.055 (3)	−0.047 (5)	−0.001 (4)	0.018 (3)
C5A	0.094 (6)	0.085 (5)	0.054 (3)	−0.033 (4)	−0.023 (4)	0.026 (3)
C6A	0.053 (4)	0.074 (4)	0.060 (3)	−0.017 (4)	−0.015 (3)	0.011 (3)
C1B	0.048 (4)	0.054 (5)	0.037 (4)	−0.023 (3)	−0.004 (3)	0.004 (3)
C2B	0.055 (4)	0.061 (4)	0.059 (4)	−0.018 (3)	−0.009 (3)	0.006 (3)
C3B	0.063 (5)	0.085 (7)	0.054 (4)	−0.033 (4)	0.007 (4)	0.000 (4)
C4B	0.084 (7)	0.086 (5)	0.043 (2)	−0.043 (5)	0.000 (3)	0.011 (3)
C5B	0.073 (5)	0.077 (5)	0.059 (3)	−0.029 (3)	−0.015 (3)	0.020 (3)
C6B	0.046 (4)	0.064 (4)	0.049 (3)	−0.017 (3)	−0.012 (3)	0.015 (3)
C7	0.0489 (7)	0.0559 (7)	0.0442 (7)	−0.0199 (6)	−0.0111 (5)	0.0075 (6)
C8	0.0723 (9)	0.0686 (9)	0.0588 (8)	−0.0265 (8)	−0.0104 (7)	−0.0072 (7)
C9	0.0963 (12)	0.0799 (11)	0.0877 (11)	−0.0487 (10)	−0.0349 (9)	0.0012 (9)
C10	0.0707 (11)	0.1179 (16)	0.0942 (13)	−0.0531 (11)	−0.0211 (8)	0.0259 (11)
C11	0.0581 (10)	0.1348 (17)	0.0706 (11)	−0.0379 (11)	−0.0002 (8)	0.0018 (11)
C12	0.0593 (9)	0.0915 (11)	0.0512 (8)	−0.0285 (8)	−0.0061 (7)	−0.0069 (8)
C13	0.0456 (7)	0.0576 (7)	0.0423 (6)	−0.0187 (6)	−0.0078 (5)	0.0064 (6)
C14	0.0693 (9)	0.0642 (8)	0.0547 (8)	−0.0355 (7)	−0.0186 (7)	0.0207 (7)
C15	0.0609 (8)	0.0604 (8)	0.0556 (8)	−0.0324 (7)	−0.0139 (6)	0.0103 (6)
C16	0.0435 (7)	0.0483 (7)	0.0447 (7)	−0.0130 (5)	−0.0059 (5)	0.0025 (5)
C17	0.0608 (8)	0.0481 (7)	0.0515 (7)	−0.0196 (6)	−0.0139 (6)	0.0104 (6)
C18	0.0600 (8)	0.0525 (7)	0.0537 (7)	−0.0269 (6)	−0.0133 (6)	0.0062 (6)
C19	0.0506 (7)	0.0526 (7)	0.0508 (7)	−0.0172 (6)	−0.0103 (6)	0.0021 (6)
C20	0.0505 (7)	0.0541 (7)	0.0480 (7)	−0.0166 (6)	−0.0102 (6)	0.0024 (6)
C21	0.0609 (8)	0.0593 (8)	0.0529 (8)	−0.0220 (7)	−0.0142 (6)	0.0026 (6)
C22	0.0500 (7)	0.0549 (7)	0.0441 (7)	−0.0128 (6)	−0.0082 (6)	−0.0011 (6)
C23	0.0801 (10)	0.0591 (8)	0.0530 (8)	−0.0257 (7)	−0.0204 (7)	−0.0014 (7)
C24	0.0892 (11)	0.0632 (9)	0.0493 (8)	−0.0209 (8)	−0.0286 (8)	−0.0015 (7)
C25	0.0655 (9)	0.0506 (7)	0.0397 (7)	−0.0065 (6)	−0.0063 (6)	−0.0026 (6)
C26	0.0573 (8)	0.0559 (8)	0.0566 (8)	−0.0179 (6)	−0.0116 (6)	0.0030 (6)
C27	0.0522 (8)	0.0616 (8)	0.0520 (7)	−0.0171 (6)	−0.0169 (6)	0.0041 (6)
C28	0.0854 (11)	0.0581 (8)	0.0522 (8)	−0.0145 (8)	0.0022 (7)	0.0032 (7)
C29	0.0617 (9)	0.0562 (8)	0.0561 (8)	−0.0176 (7)	−0.0079 (7)	0.0037 (7)
C30	0.0855 (12)	0.0692 (10)	0.0657 (10)	−0.0209 (9)	−0.0041 (8)	−0.0113 (8)
N1	0.0544 (7)	0.0804 (8)	0.0422 (6)	−0.0327 (6)	−0.0109 (5)	0.0138 (6)
O1	0.1428 (12)	0.0904 (8)	0.0880 (9)	−0.0721 (9)	−0.0625 (8)	0.0315 (7)
O2	0.1030 (8)	0.0547 (6)	0.0473 (5)	−0.0146 (6)	−0.0214 (5)	0.0021 (4)

### (2E)-3-[4-(Diphenylamino)phenyl]-1-[4-(prop-1-yn-2-yloxy)phenyl]prop-2-en-1-one Geometric parameters (Å, º)

**Table:**

C1A—C2A	1.382 (8)	C13—C14	1.3822 (18)
C1A—C6A	1.385 (9)	C13—C18	1.3862 (18)
C1A—N1	1.405 (11)	C13—N1	1.4106 (16)
C2A—C3A	1.372 (8)	C14—C15	1.3792 (19)
C2A—H2A	0.9300	C14—H14	0.9300
C3A—C4A	1.361 (7)	C15—C16	1.3848 (18)
C3A—H3A	0.9300	C15—H15	0.9300
C4A—C5A	1.373 (7)	C16—C17	1.3924 (18)
C4A—H4A	0.9300	C16—C19	1.4552 (17)
C5A—C6A	1.392 (9)	C17—C18	1.3752 (18)
C5A—H5A	0.9300	C17—H17	0.9300
C6A—H6A	0.9300	C18—H18	0.9300
C1B—C6B	1.372 (10)	C19—C20	1.3230 (18)
C1B—C2B	1.383 (10)	C19—H19	0.9300
C1B—N1	1.454 (13)	C20—C21	1.4697 (18)
C2B—C3B	1.389 (9)	C20—H20	0.9300
C2B—H2B	0.9300	C21—O1	1.2161 (17)
C3B—C4B	1.371 (8)	C21—C22	1.4856 (19)
C3B—H3B	0.9300	C22—C27	1.3839 (19)
C4B—C5B	1.382 (8)	C22—C23	1.3940 (18)
C4B—H4B	0.9300	C23—C24	1.367 (2)
C5B—C6B	1.367 (10)	C23—H23	0.9300
C5B—H5B	0.9300	C24—C25	1.379 (2)
C6B—H6B	0.9300	C24—H24	0.9300
C7—C8	1.3775 (19)	C25—O2	1.3658 (16)
C7—C12	1.381 (2)	C25—C26	1.3792 (19)
C7—N1	1.4215 (16)	C26—C27	1.3815 (19)
C8—C9	1.390 (2)	C26—H26	0.9300
C8—H8	0.9300	C27—H27	0.9300
C9—C10	1.365 (3)	C28—O2	1.4271 (19)
C9—H9	0.9300	C28—C29	1.454 (2)
C10—C11	1.356 (3)	C28—H28A	0.9700
C10—H10	0.9300	C28—H28B	0.9700
C11—C12	1.369 (2)	C29—C30	1.162 (2)
C11—H11	0.9300	C30—H30	0.9300
C12—H12	0.9300		
			
C2A—C1A—C6A	117.4 (8)	C15—C14—C13	120.72 (13)
C2A—C1A—N1	122.0 (9)	C15—C14—H14	119.6
C6A—C1A—N1	120.6 (8)	C13—C14—H14	119.6
C3A—C2A—C1A	121.1 (7)	C14—C15—C16	121.60 (12)
C3A—C2A—H2A	119.5	C14—C15—H15	119.2
C1A—C2A—H2A	119.5	C16—C15—H15	119.2
C4A—C3A—C2A	120.9 (6)	C15—C16—C17	117.20 (11)
C4A—C3A—H3A	119.6	C15—C16—C19	119.95 (12)
C2A—C3A—H3A	119.6	C17—C16—C19	122.83 (12)
C3A—C4A—C5A	120.0 (6)	C18—C17—C16	121.44 (12)
C3A—C4A—H4A	120.0	C18—C17—H17	119.3
C5A—C4A—H4A	120.0	C16—C17—H17	119.3
C4A—C5A—C6A	119.0 (6)	C17—C18—C13	120.78 (12)
C4A—C5A—H5A	120.5	C17—C18—H18	119.6
C6A—C5A—H5A	120.5	C13—C18—H18	119.6
C1A—C6A—C5A	121.7 (7)	C20—C19—C16	127.70 (13)
C1A—C6A—H6A	119.2	C20—C19—H19	116.1
C5A—C6A—H6A	119.2	C16—C19—H19	116.1
C6B—C1B—C2B	121.3 (9)	C19—C20—C21	121.40 (13)
C6B—C1B—N1	119.6 (9)	C19—C20—H20	119.3
C2B—C1B—N1	119.0 (10)	C21—C20—H20	119.3
C1B—C2B—C3B	119.7 (8)	O1—C21—C20	120.04 (13)
C1B—C2B—H2B	120.1	O1—C21—C22	119.93 (12)
C3B—C2B—H2B	120.1	C20—C21—C22	120.02 (12)
C4B—C3B—C2B	118.9 (7)	C27—C22—C23	117.38 (13)
C4B—C3B—H3B	120.6	C27—C22—C21	123.91 (12)
C2B—C3B—H3B	120.6	C23—C22—C21	118.70 (13)
C3B—C4B—C5B	120.4 (7)	C24—C23—C22	121.24 (14)
C3B—C4B—H4B	119.8	C24—C23—H23	119.4
C5B—C4B—H4B	119.8	C22—C23—H23	119.4
C6B—C5B—C4B	121.2 (8)	C23—C24—C25	120.35 (13)
C6B—C5B—H5B	119.4	C23—C24—H24	119.8
C4B—C5B—H5B	119.4	C25—C24—H24	119.8
C5B—C6B—C1B	118.4 (9)	O2—C25—C24	115.39 (12)
C5B—C6B—H6B	120.8	O2—C25—C26	124.74 (13)
C1B—C6B—H6B	120.8	C24—C25—C26	119.88 (13)
C8—C7—C12	118.85 (13)	C25—C26—C27	119.20 (13)
C8—C7—N1	121.16 (13)	C25—C26—H26	120.4
C12—C7—N1	119.99 (12)	C27—C26—H26	120.4
C7—C8—C9	119.40 (15)	C26—C27—C22	121.95 (12)
C7—C8—H8	120.3	C26—C27—H27	119.0
C9—C8—H8	120.3	C22—C27—H27	119.0
C10—C9—C8	120.86 (15)	O2—C28—C29	112.91 (13)
C10—C9—H9	119.6	O2—C28—H28A	109.0
C8—C9—H9	119.6	C29—C28—H28A	109.0
C11—C10—C9	119.46 (16)	O2—C28—H28B	109.0
C11—C10—H10	120.3	C29—C28—H28B	109.0
C9—C10—H10	120.3	H28A—C28—H28B	107.8
C10—C11—C12	120.68 (17)	C30—C29—C28	178.59 (17)
C10—C11—H11	119.7	C29—C30—H30	180.0
C12—C11—H11	119.7	C1A—N1—C13	117.1 (6)
C11—C12—C7	120.73 (15)	C1A—N1—C7	123.6 (6)
C11—C12—H12	119.6	C13—N1—C7	119.28 (10)
C7—C12—H12	119.6	C13—N1—C1B	124.9 (8)
C14—C13—C18	118.26 (12)	C7—N1—C1B	115.7 (7)
C14—C13—N1	121.10 (12)	C25—O2—C28	118.79 (11)
C18—C13—N1	120.64 (12)		
			
C6A—C1A—C2A—C3A	−0.7 (13)	C19—C20—C21—C22	−174.11 (12)
N1—C1A—C2A—C3A	176.0 (15)	O1—C21—C22—C27	179.55 (15)
C1A—C2A—C3A—C4A	−1.0 (11)	C20—C21—C22—C27	−1.0 (2)
C2A—C3A—C4A—C5A	1.9 (15)	O1—C21—C22—C23	−2.2 (2)
C3A—C4A—C5A—C6A	−1.0 (16)	C20—C21—C22—C23	177.29 (13)
C2A—C1A—C6A—C5A	2 (2)	C27—C22—C23—C24	0.6 (2)
N1—C1A—C6A—C5A	−175.2 (14)	C21—C22—C23—C24	−177.77 (14)
C4A—C5A—C6A—C1A	−1 (2)	C22—C23—C24—C25	−0.7 (2)
C6B—C1B—C2B—C3B	0.2 (16)	C23—C24—C25—O2	−179.69 (13)
N1—C1B—C2B—C3B	−178.2 (18)	C23—C24—C25—C26	0.4 (2)
C1B—C2B—C3B—C4B	0.1 (12)	O2—C25—C26—C27	−179.91 (13)
C2B—C3B—C4B—C5B	−0.1 (17)	C24—C25—C26—C27	0.0 (2)
C3B—C4B—C5B—C6B	−0.2 (19)	C25—C26—C27—C22	−0.1 (2)
C4B—C5B—C6B—C1B	0 (2)	C23—C22—C27—C26	−0.2 (2)
C2B—C1B—C6B—C5B	−1 (2)	C21—C22—C27—C26	178.08 (13)
N1—C1B—C6B—C5B	177.9 (16)	C2A—C1A—N1—C13	39.6 (15)
C12—C7—C8—C9	−0.3 (2)	C6A—C1A—N1—C13	−143.8 (12)
N1—C7—C8—C9	179.61 (14)	C2A—C1A—N1—C7	−139.5 (9)
C7—C8—C9—C10	−0.7 (3)	C6A—C1A—N1—C7	37.1 (19)
C8—C9—C10—C11	0.9 (3)	C14—C13—N1—C1A	38.7 (8)
C9—C10—C11—C12	−0.1 (3)	C18—C13—N1—C1A	−141.4 (8)
C10—C11—C12—C7	−0.9 (3)	C14—C13—N1—C7	−142.17 (14)
C8—C7—C12—C11	1.1 (2)	C18—C13—N1—C7	37.67 (19)
N1—C7—C12—C11	−178.83 (15)	C14—C13—N1—C1B	33.6 (10)
C18—C13—C14—C15	0.0 (2)	C18—C13—N1—C1B	−146.6 (9)
N1—C13—C14—C15	179.86 (13)	C8—C7—N1—C1A	44.8 (9)
C13—C14—C15—C16	0.2 (2)	C12—C7—N1—C1A	−135.3 (9)
C14—C15—C16—C17	−0.4 (2)	C8—C7—N1—C13	−134.28 (14)
C14—C15—C16—C19	−178.66 (13)	C12—C7—N1—C13	45.62 (19)
C15—C16—C17—C18	0.4 (2)	C8—C7—N1—C1B	49.6 (9)
C19—C16—C17—C18	178.59 (13)	C12—C7—N1—C1B	−130.5 (9)
C16—C17—C18—C13	−0.2 (2)	C6B—C1B—N1—C13	−136.2 (14)
C14—C13—C18—C17	0.0 (2)	C2B—C1B—N1—C13	42.2 (17)
N1—C13—C18—C17	−179.88 (12)	C6B—C1B—N1—C7	40 (2)
C15—C16—C19—C20	177.62 (13)	C2B—C1B—N1—C7	−141.9 (10)
C17—C16—C19—C20	−0.6 (2)	C24—C25—O2—C28	178.75 (13)
C16—C19—C20—C21	−178.67 (13)	C26—C25—O2—C28	−1.4 (2)
C19—C20—C21—O1	5.4 (2)	C29—C28—O2—C25	70.02 (17)

### (2E)-3-[4-(Diphenylamino)phenyl]-1-[4-(prop-1-yn-2-yloxy)phenyl]prop-2-en-1-one Hydrogen-bond geometry (Å, º)

**Table:**

*D*—H···*A*	*D*—H	H···*A*	*D*···*A*	*D*—H···*A*
C30—H30···O1^i^	0.93	2.55	3.054 (2)	114
C28—H28*A*···*Cg*(C7–C12)^ii^	0.93	2.97	3.853 (2)	153
C30—H30···*Cg*(C22–C27)^iii^	0.93	2.72	3.527 (2)	145

Symmetry codes: (i) *x*, *y*−1, *z*; (ii) *x*−1, *y*+1, *z*−1; (iii) −*x*, −*y*+2, −*z*.
